# Variable selection under multiple imputation using the bootstrap in a prognostic study

**DOI:** 10.1186/1471-2288-7-33

**Published:** 2007-07-13

**Authors:** Martijn W Heymans, Stef van Buuren, Dirk L Knol, Willem van Mechelen, Henrica CW de Vet

**Affiliations:** 1 Vrije Universiteit, Institute for Health Sciences, Department of Methodology and Applied Biostatistics, Amsterdam, The Netherlands; 2 Body@Work, Research Center Physical Activity, Work and Health, TNO-VUmc, Amsterdam, The Netherlands; 3 Department of Public and Occupational health, VU University Medical Center, Amsterdam, The Netherlands; 4 Institute for Research in Extramural Medicine, VU University Medical Center, Amsterdam, The Netherlands; 5 TNO Quality of Life, Leiden, The Netherlands; 6 Department of Methodology and Statistics, University of Utrecht, The Netherlands; 7 Clinical Epidemiology and Biostatistics, VU University Medical Center, Amsterdam, The Netherlands; 8 EMGO-Institute (Metropolitan building), VU University Medical Center, Van der Boechorststraat 7, 1081 BT Amsterdam, The Netherlands

## Abstract

**Background:**

Missing data is a challenging problem in many prognostic studies. Multiple imputation (MI) accounts for imputation uncertainty that allows for adequate statistical testing. We developed and tested a methodology combining MI with bootstrapping techniques for studying prognostic variable selection.

**Method:**

In our prospective cohort study we merged data from three different randomized controlled trials (RCTs) to assess prognostic variables for chronicity of low back pain. Among the outcome and prognostic variables data were missing in the range of 0 and 48.1%. We used four methods to investigate the influence of respectively sampling and imputation variation: MI only, bootstrap only, and two methods that combine MI and bootstrapping. Variables were selected based on the inclusion frequency of each prognostic variable, i.e. the proportion of times that the variable appeared in the model. The discriminative and calibrative abilities of prognostic models developed by the four methods were assessed at different inclusion levels.

**Results:**

We found that the effect of imputation variation on the inclusion frequency was larger than the effect of sampling variation. When MI and bootstrapping were combined at the range of 0% (full model) to 90% of variable selection, bootstrap corrected c-index values of 0.70 to 0.71 and slope values of 0.64 to 0.86 were found.

**Conclusion:**

We recommend to account for both imputation and sampling variation in sets of missing data. The new procedure of combining MI with bootstrapping for variable selection, results in multivariable prognostic models with good performance and is therefore attractive to apply on data sets with missing values.

## Background

The development of chronic low back pain is an important societal problem. From a prevention perspective, it is necessary to identify as early as possible the patients that are at high risk for developing chronic low back pain and long-term disability. This study aims to investigate the variable selection process in a prognostic model for high risk patients using merged data from three different studies [1-3]. Patients with low back pain were enrolled in each study, and similar baseline and follow-up information was measured. As some variables were measured in only one or two studies, merging the studies resulted in high percentages of missing values for these data. Discarding these prognostic variables would undermine the validity of the models. This study therefore set outs to develop a prognostic model from incomplete data.

The presence of missing data is a frequently encountered problem in the development of prognostic models. The default strategy is to eliminate all incomplete cases from the analysis. As the amount of incomplete cases can rapidly increase with the number of variables considered, this strategy is wasteful of costly collected data [4]. Single imputation, such as mean imputation or imputation based on linear regression, leads to incorrect statistical tests because the complete-data analysis does not account for uncertainty created by the fact that data are missing [5,6]. Multiple imputation (MI) accounts for the uncertainty caused by the missing data, and when properly done, MI provides correct statistical inferences [6]. MI replaces each missing values by two or more imputations. The spread between the imputed values reflects the uncertainty about the missing data. MI proceeds by applying the complete-data analysis to each imputed data set, followed by pooling the results into a final estimate. Such pooling is usually straightforward, but introduces complexities if automatic variable selection strategies are applied. The variable selection algorithm may easily produce different models for different imputed data sets. Some authors suggested including variables into the common model that appear in at least 3 out of 5 (60%) of the model [7,8], and pool these coefficients. Some simulation work has been done with encouraging results, but applications in using MI in prognostic modeling are still rare [9].

Model building in prognostic studies is often conducted by automatic backward or forward selection procedures. It is well known that stepwise methods have disadvantages: their power to select true variables is limited, they may include noise variables in the final model, they may lead to biased regression coefficients and to overly optimistic estimates of predictive ability and model fit, and the set of predictors may be unstable [10,11]. For example, in a simulated case-control study using stepwise regression of variables declared to be significant with *p*-values between 0.01 and 0.05, only 49 percent were true risk factors [12]. Model selection problems occur if the optimal model in the available sample is different from the optimal model in the population of interest. Problems grow as the sample size becomes smaller.

Improving the methodology for stepwise model building is an active and rapidly expanding research area in both theoretical and applied statistics. In the sequel, we will focus on particular line of research that combines bootstrapping with automatic backward regression [13-16]. This methodology randomly draws multiple samples with replacement from the observed sample, thus mimicking the sampling variation in the population from which the sample was drawn. Stepwise regression analyses are then performed on each bootstrap sample. The proportion of times that each prognostic variable is retained into the stepwise regression model provides information about the strength of the evidence that an indicator is an independent predictor. Variables that have a strong effect on the outcome will be included more frequently than variables with no or a weak effect [13]. The hope is that the model derived when variables are included in this way is closer to the optimal model in the population. Using similar technology, it is also possible to measure and correct for overly optimistic inferences obtained from analysing a single data set. See Harrell and Steyerberg [11,17] for an overview.

There are interesting similarities between the missing data and the bootstrap regression modeling methodologies. Both replicate the key variation into multiple data sets, analyse each data set separately, and synthesize the replicated analyses into a final inference. For missing data, the key variation consists of the spread of the multiply imputed values. In bootstrap regression modeling, the relevant variation derives from the fact that only one sample is available. Both sources of variation complicate prognostic model building, and both may cause biased, inefficient or overly optimistic model predictions. The objective of this paper is to examine and correct for the influence of both sources of variation, i.e., variation induced by sampling as well as extra variation created by incomplete data. Both MI and bootstrapping generate multiple datasets. The main purpose of this article is to examine how MI and bootstrapping can be properly combined into the selection process of prognostic variables if there is missing data and to examine if this influences model performance.

## Methods

### Study design and population

A prospective cohort study design was formed by merging data from three recent randomised controlled trials in low back pain patients. The first trial determined the effectiveness of a behaviorally oriented graded activity program in comparison to usual care (134 patients) [1]. The second trial compared participative ergonomics interventions and graded activity to usual care (195 patients) [2]. The third trial compared high and low intensity back schools with usual care (299 patients) [3]. Consequently, the study population consists of 628 patients in total. All patients visited their occupational physician (OP) at one of the participating Occupational Health Services (OHS) when they were on sick-leave because of low back pain for not more than 8 weeks. All studies had a follow-up of at least one year.

### Outcome measures

The study was aimed to assess prognostic variables for chronic low back pain. We defined the outcome chronic low back pain as having pain indicated with a minimum score of ≥1 on the VAS scale (0–10) measured at baseline, 12 and 26 weeks follow-up. The potential prognostic variables were assessed by means of self-reported questionnaires before inclusion in the studies.

### Potential prognostic variables

The following variables were considered important: age, gender, duration of current episode of low back pain, radiation to one or both legs, treatment during study enrollment, education level, quality of life and body mass index [18-20]. Also pain intensity at baseline measured by the VAS scale [21] and functional status at baseline measured by the Roland Disability Questionnaire (RDQ) [22] were assessed. To asses short-term change in pain intensity and in functional status we performed calculations based on change scores of pain and RDQ respectively between baseline and 26 weeks follow-up. We also included the absolute level of RDQ score achieved at 26 weeks follow-up. Work-related physical variables were measured by the section 'musculoskeletal workload' of the Dutch Musculoskeletal Questionnaire (DMQ) [23]. These variables are daily exposition to sitting, stooping, lifting, whole body vibration, working with vibration tools, working with hands under knee level, bending and twisting of the trunk. Physical activity was measured with the Baecke questionnaire [24] and height and weight were used to calculate the Body Mass Index (BMI). The presence of full or partial work absence at baseline was also identified. Work-related psychosocial variables were measured by means of a Dutch version of the Job Content Questionnaire. Dimensions distinguished by this questionnaire are: quantitative job demands, job control and social support [25]. Job satisfaction was assessed by means of a question concerning job task satisfaction [26]. Self-prediction concerning the timing of return to work was also assessed. The extent to which people feared that exercise could lead to reinjury was measured with the Dutch version of the Tampa Scale for Kinesiophobia [27]. Fear of movement, avoidance of activities and back pain beliefs were measured with the Fear Avoidance Beliefs Questionnaire [28]. Active and passive coping with pain was measured with the Pain Coping Inventory Scale [29].

### Statistical analyses

The objective was to construct a prognostic model under imputation and sampling variation 1) that used all available information and 2) that has been corrected for any optimism arising from the model selection process. In order to achieve this goal, we multiply imputed the merged data sets 100 times. For each imputed data set, we constructed 200 bootstrap data sets by randomly drawing with replacement. The total number of data sets was thus equal to 100 (number of imputations) times 200 (number of bootstrap samples) is 20,000 data sets. Automatic backward logistic regression analyses were used at various levels of this nested procedure, according to four methods:

#### Imputation (MI)

automatic backward selection was applied to on 100 imputed datasets. Any differences between the 100 models is due to the uncertainty created by the missing data.

#### Bootstrapping (B)

automatic backward selection was applied by drawing 200 bootstrap samples from the first imputed dataset only. Any differences between these 200 models is due to model uncertainty created by sampling.

#### MI100+B

automatic backward selection was applied to the 20,000 data sets from the nested procedure. Any differences between these 20,000 models is due to uncertainty created both the missing data and the sample design.

#### MI10+B

automatic backward selection was applied to the first 2,000 data sets from *M100+B *method. The results from this method explores whether generation of 10 multiple imputed data sets, which is commonly used within the MI framework, will yield the same results as the *MI100+B *method.

It has to be noted here that is not our purpose to define B as a separate prognostic modeling method. We present both the B and MI method to identify the amount of variation generated by each method. By reporting the MI method and not reporting the B method, it would be impossible to explain where the major variation occurred in the MI + B100 and MI + B10 methods. Therefore we choose to report on the variation induced by each method.

### Complete-data model

All automatic stepwise methods used a probability to remove of 0.5. Although this value seems relatively high, it is favorable for backward logistic regression analyses [10]. To explore the sensitivity of this selection criterion we repeated variable selection with a p-value of 0.157 [30]. All regression models adjusted for the effects of the interventions by including the variable treatment into the models. In all models chronic pain was fitted as the dependent variable and the prognostic indicators as the independent variables. The number of events per variable in these models was 4.4.

### Imputation of the missing data

Variables with incomplete baseline and follow-up data were completed by multiple imputation (MI). As not all trials collected all prognostic variables, it is reasonable to assume that the data are Missing at Random (MAR). MI was performed with the Multivariate Imputation by Chained Equations (MICE) procedure [31]. This is a flexible imputation method which allows one to specify the multivariate structure in the data as a series of conditional imputation models. Logistic regression is used to impute incomplete dichotomous variables, linear regression to impute continues variables.

Specification of the imputation model was done according to the guidelines described by Van Buuren et al [32] and Clark and Altman [9]. As a starting point we included all 31 variables that would be used in the full multivariable model. Due to multicolinearity and computational problems it was not feasible to run this model. We therefore refined the imputation model. We kept all complete variables into the model and further maximized the number of variables on basis of their correlation (> 0.2) with the variables to be imputed. This resulted in a series of imputation models that consisted of the best 10 to 25 predictor variables.

MICE was done with the closest predictor option ("predictive mean matching") as described in Rubin [6] and Van Buuren et al. [32]. This method models a missing variable Y as a linear combination of predictor variables X, finds the complete case whose Y estimate is closest to that of the current incomplete case, and takes the observed Y from the former as the imputed Y value for the latter. With this approach only a subset of the predictor values is used to find the complete case which makes this procedure robust against non-normal linear combinations. We constructed 100 imputed datasets. This high value was chosen in order to be able to estimate the inclusion frequency per variable (see next paragraph) in the final model with adequate precision.

### Selection of prognostic indicators by bootstrapping

In each of the four methods described above we calculated the proportion of times that a variable appears in the models and call this number the inclusion frequency [13]. The final model under each method was estimated by 1) taking up the predictors whose inclusion frequency exceeds a certain threshold, and 2) estimating the regression weights of this predictor set on the imputed data. We chose the following threshold values: 0.0 (the full model), 0.6, 0.7, 0.8 and 0.9 (the smallest model).

### Model performance

The performance of the models developed by the four methods was explored in terms of their discriminative and calibrative ability. The discriminative ability was measured by the so-called *c*-index, which is equal to the area under the curve (AUC) for logistic models and was corrected, by using bootstrapping, for optimism in the original sample [33]. The idea is as follows. A stepwise model is fitted on each bootstrap sample, and that model is used to calculate the *c*-index in the original sample. The bootstrap model will typically be less successful than a model optimized on the original sample. The size of the difference in the *c*-index between the bootstrap sample and the original sample indicates the amount of optimism in the original model. The *c*-index is an index of predictive strength that measures how well the model can distinguish between patients with a high and low risk for chronic low back pain. A higher *c*-index means a model with a better discriminative ability with perfect discrimination with a c-index of 1. Calibration of the models is considered by calculating the slope of the prognostic index (PI). The slope can be considered a correction factor for too optimistic regression estimates in the original sample and is also calculated by using bootstrapping. The size of the difference in predictability, of the PI fitted on the bootstrap sample and on the original sample, indicates the amount of optimism of the original model. A slope of < 1 indicates that low predictions for chronic low back pain are too low and high predictions are too high, which means that the model is too optimistic [34]. For the c-index and the slope 200 bootstrap samples were used.

### Software

The MICE imputation procedure [35] as well as the backward selection procedure were performed with S-Plus software (version 2000). We developed additional S-plus routines to perform bootstrap selection and to evaluate model performance building on the MICE and Design Libraries [36].

## Results

Table 1 gives an overview of the percentage of missing data and some summary statistics of the data. The variables physical activity and fear avoidance beliefs contain high percentages of missing data (44.6% and 48.1% respectively). Other variables had missing values within the range of 0 – 33.3%. The overall number of chronic low back pain patients is 493 (of out 628), which is a prevalence of 79%. For the individual studies the prevalence rates were: trial 1: 111 (of out 134) patients (83%); trial 2: 139 (of out 195) patients (71%) and trial 3: 243 (of out 299) patients (81%) with chronic low back pain. Table 2 lists the inclusion frequencies of prognostic variables selected by the four methods. A value of 100% means that the indicator was selected in each replication. Table 2 shows that both the inclusion frequency and the sequence in which indicators were retained varied considerably between MI and B. For MI, the inclusion frequency varied from 27% to 100%, whereas that range was 51.8% to 100% for B. This indicates that MI is more sensitive to distinguish variables with a strong effect from variables with a weak effect on the outcome. With respect to the sequence of prognostic indicators, three (level of functional status at 3 months, change in pain intensity and pain at baseline) and two indicators (level of functional status at 3 months and change in pain intensity) were selected in 100% of the samples by methods MI and B respectively. Note that about 20% of these variables were missing, so there is substantial potential for missing data variation in the imputed data. It is reassuring to see that MI imputes data in such a way that the most important predictors are retained. Also, physical activity had 44% missing data. Its inclusion frequency under MI (95%) is considerably lower than under B (99.4%), but still very high. Agreement is generally less at lower levels of inclusion frequencies.

**Table 1:** Patient characteristics and missing data information (n = 628)

	**Trial 1 (n = 134)**	**Trial 2 (n = 195)**	**Trial 3 (n = 299)**	**Missing (%)**	**Value**
Age (mean years ± sd)				0	40.6 (9.5)
Gender (male, %)				0	71.0
Education (%)				26.1	73.9
Smoking (%)				7.5	43.8
Self-predicted certainty at 6 months (%)				24.4	75.6
Physical activity (mean ± sd)				44.6	8.8 (1.0)
Bodyweight (kg) (mean ± sd)				3.3	81.1 (15.8)
Height (cm) (mean ± sd)				3.3	176.6 (9.4)
Quality of life (mean ± sd)				2.7	0.5 (0.07)
Years working in current job (median, IQR)				24.2	35.0 (28)
Full work absence (vs partial) (%)				0.8	67.0
Job satisfaction (%)				2.9	97.1
Job Content Questionnaire:					
Job control (mean ± sd)				25.6	56.2 (9.2)
Job demands (mean ± sd)				24.7	33.1 (4.8)
Social support (mean ± sd)				24.8	22.5 (4.1)
Daily exposed to:					
Vibration tools (%)				27.1	5.7
Lifting >25 kg (%)				24.2	15.3
Bending and twisting of the trunk (%)				24.2	20.2
Whole body vibration (%)				26.4	7.8
Sitting (%)				24.7	14.2
Working with hands under knee level (%)				25.0	6.6
Stooping (%)				25.2	19.6
Duration of complaints (weeks) prior to randomization; (median, IQR)				33.3	5.8 (13.3)
Pain radiation in 1 or both legs (%)				2.1	33.8
Functional status (RDQ) (mean ± sd)				5.1	11.3 (5.2)
Pain intensity (VAS) in (mean ± sd)				3.0	6.2 (1.9)
Treatment during enrollment (%)				23.6	76.7
Pain intensity at 3 months (VAS) (mean ± sd)				17.8	4.5 (2.5)
Functional status at 3 months (RDQ) (mean ± sd)				19.9	8.8 (6.1)
Change in pain intensity* (VAS) (mean ± sd)				20.0	2.2 (2.8)
Pain coping, active (mean ± sd)				5.6	6.7 (1.2)
Pain coping, passive (mean ± sd)				7.0	6.5 (1.3)
Fear avoidance beliefs (FAB) (mean ± sd)				48.1	19.5 (9.7)
Kinesiophobia (Tampa) (mean ± sd)				6.2	39.8 (6.7)
Body mass index				4.1	25.9 (4.0)

* Average of prognostic indicators representing change in pain.

**Table 2:** Inclusion frequencies and average rank per indicator selected by the four methods (MI = multiple imputations, B = bootstrap, M100+B = 100 imputations+bootstap, MI10+B = 10 imputations+bootstrap)

	Method									
	MI†		B†		MI100+B†		MI10+B†		MI10 + B‡	

	%	rank	%	rank	%	rank	%	rank	%	rank

Level of functional status at 3 months	100.0	1	100.0	1	99.4	1	99.5	1	88.0	3
Change in pain intensity	100.0	2	100.0	2	99.3	2	98.5	2	99.1	1
Pain at baseline	100.0	3	90.2	6	96.2	3	95.7	3	97.7	2
Physical activity	95.0	4	99.4	3	85.7	4	91.2	4	61.3	5
Vibration tools	90.0	5	94.2	4	81.0	5	80.9	5	43.7	13
Whole body vibration	88.0	6	71.0	12	77.2	7	79.6	6	50.2	4
Sitting	86.0	7	75.6	10	76.5	9	75.7	9	50.2	9
Job demands	81.0	8	65.6	16	77.7	6	79.0	7	52.2	8
Passive pain coping	77.0	9	51.8	30	71.8	11	72.5	11	41.1	15
Duration of complaints	70.0	10	71.2	11	76.5	8	77.4	8	35.9	19
Body mass index	63.0	11	90.8	5	66.1	13	69.0	14	39.2	16
Treatment during enrollment	62.0	12	85.8	7	75.5	10	75.5	10	46.2	11
Pain radiation	61.0	13	85.8	8	68.2	12	69.4	12	31.6	21
Working with hands under knee level	59.0	14	76.8	9	65.9	15	68.1	15	31.2	22
Education level	57.0	15	66.0	15	65.7	14	65.1	16	32.1	20
Job control	53.0	16	64.2	17	65.4	16	69.1	13	36.9	18
Quality of life	51.0	17	51.8	31	62.5	21	60.8	23	24.9	26
Bending and twisting of the trunk	50.0	18	56.8	22	64.6	17	62.9	17	45.9	12
Age	48.0	19	68.4	13	62.2	22	57.5	29	23.0	29
Lifting	48.0	20	66.6	14	61.4	24	60.4	25	41.8	14
Fear avoidance beliefs	48.0	21	55.8	26	63.3	19	61.8	21	25.4	25
Change in functional status	44.0	22	52.0	29	63.4	18	59.9	26	55.9	6
Kinesiophobia	43.0	23	52.8	28	63.2	20	60.4	24	22.6	30
Gender	42.0	24	56.2	24	61.5	23	61.5	20	24.6	27
Social support	41.0	25	56.0	25	60.0	26	62.8	18	27.9	24
Self-predicted certainty at 6 months	35.0	26	61.6	18	60.9	25	62.5	19	28.1	23
Active pain coping	32.0	27	54.4	27	59.9	27	58.6	28	23.5	28
Functional status at baseline	31.0	28	58.0	21	59.1	28	57.0	30	46.8	10
Stooping	30.0	29	58.8	19	57.7	30	59.4	27	53.0	7
Job satisfaction	29.0	30	58.4	20	58.2	29	60.9	22	37.7	17
Work absence at baseline	27.0	31	56.8	23	57.1	31	55.1	31	19.2	31

Rank: the sequence of indicators in order of their appearance into the backward regression models.

%: the proportion of times that the indicator is retained in the backward regression models (inclusion frequency).

† P-value used of 0.5.

‡ P-value used of 0.157.

At a threshold level of 60%, MI selects 13 prognostic variables while B selects 18 variables, The combined methods (MI100+B, MI10+B) agree quite well with each other, and tend to be similar to method B in terms of the range of absolute inclusion frequency. However, the sequence of variables is more closely related to MI, indicating that the missing data variation has more influence on the inclusion sequence than the sampling variation.

When MI10+B is applied using a stricter p-value of 0.157, step 1 selects variables having inclusion frequencies between 19.2 to 99.1. Using a cut off value of 80% for the inclusion frequency, resulted in a model in which the first 3 variables agreed.

Table 3 summarizes the performance of the models developed by the four methods where the p-value of 0.5 was used. It presents the number of variables included in the full model, and at inclusion levels of at least 90, 80, 70 and 60%. It also provides the corresponding discriminative and calibrative indices, respectively the *c*-index and the slope of the PI.

**Table 3:** The performance of methods 1 to 4 at different levels of proportions of selected indicators

	MI†				B†				MI100+BI†				MI10+BI†			
		c-index		slope		c-index		slope		c-index		slope		c-index		slope
								
Threshold	n	AP	BC	BC	n	AP	BC	BC	n	AP	BC	BC	n	AP	BC	BC

90%	5	0.74	0.71	0.86	6	0.74	0.71	0.85	3	0.74	0.71	0.86	4	0.72	0.71	0.85
80%	8	0.76	0.71	0.85	8	0.76	0.72	0.83	5	0.74	0.71	0.84	5	0.74	0.71	0.85
70%	10	0.76	0.71	0.77	12	0.78	0.72	0.80	11	0.77	0.72	0.79	11	0.77	0.72	0.80
60%	13	0.77	0.72	0.70	18	0.79	0.71	0.75	27	0.79	0.70	0.67	26	0.79	0.70	0.64
0% (full model)	31	0.80	0.70	0.65	31	0.79	0.69	0.65	31	0.80	0.70	0.64	31	0.80	0.70	0.65

n: number of indicators selected in the multivariable models.

AP: apparent index

BC: bootstrap corrected index

† Applied to the models that were developed when a p-value of 0.5 was used.

The reduced model, with prognostic indicators included whose inclusion frequency exceeds a threshold of 90% showed apparent *c*-indices between 0.72 and 0.74. The bootstrap corrected c-index was 0.71 for all four methods. These are relatively low values of the AUC, implying that these models do not distinguish patients with and without chronic low back pain very well. Including more variables into the model increases the apparent *c*-index to 0.79–0.80, but a substantial part of the apparent increase of predictability is due to model optimism. By applying the bootstrap the c-index is adjusted to 0.69 and 0.70.

The slope of the PI is 0.86 for the simplest models (threshold 90%), but drops to 0.64 for the more comprehensive models. This means that the performance in new samples is likely to be affected, and that the more elaborate models are unlikely to achieve the apparent *c*-index of 0.77–0.79 when applied in new samples.

Given these results, a parsimonious prognostic model that accounts for both the missing data and variable selection variation will 1) shrink the regression weight by a factor of 0.85–0.86 and 2) lowers the apparent *c*-index to an adjusted estimate of 0.71, i.e., the value expected when the prognostic model is applied to new data.

## Discussion

The effects of multiple imputation and bootstrapping on the inclusion frequency of prognostic indicators was investigated using four methods. For our data set, it appeared that multiple imputation lead to a relatively large spread in inclusion frequency, which is a nice property that eases decisions about which variables to include. In general, predictive models resulting from the combined methods were more similar to those generated by the imputation method than to those according to the bootstrap method. Incorporating variation from both the missing data and the model selection process revealed as much optimism as using either source alone. Optimism in the apparent c-index was larger for the more comprehensive models, i.e. when more variables are included who have a weak effect on the outcome in the models. The amount of bootstrap correction of the apparent c-index was almost independent of which sources of variation were included. This also accounted for the slope of the PI, or the amount of shrinkage needed.

It is useful to account for sampling variation by bootstrapping, and this method is slowly gaining acceptance within the research community. Our study suggests that the bootstrap method alone might not be enough if the data set contains missing values. Many clinical data sets contain substantial amounts of missing data, but the influence of missing data on the inclusion frequency of prognostic variables used to select a model is hardly recognized [37]. In contrast to the study by Clarke and Altman that had fewer missing data [9], we found a substantial effect of imputation on the apparent *c*-index estimate, especially for the more complex models. We therefore advocate the combined use of MI and bootstrapping, which addresses both imputation and sampling variation.

Note that some variables had over 45% of missing data. We nevertheless found that using 10 imputed datasets resulted in a similar selection of prognostic indicators than the use of 100 imputed datasets. This is in line with the claim of Rubin that 5 to 10 imputed datasets are enough to achieve high efficiency [6].

The bootstrap draws samples with replacement from the same data set. As was presented in table 2, the inclusion frequencies of the bootstrapping methods were less variable than those of the MI method. Thus, bootstrapping in addition to MI in our study only led to a small increase in variation of the inclusion frequency. In general, sampling variation resulting from bootstrapping varies with respect to the sample size and the number of bootstrap samples drawn. The latter number must be high enough to minimize simulation variance. By using 200 bootstrap samples simulation variance decreases as well as the bias caused by these source of variance [38].

To identify relevant prognostic variables in our study we applied automatic backward selection in combination with bootstrapping [14-17] methods which are frequently used for this purpose. It has been shown that automatic backward regression can lead to an unstable selection of prognostic indicators [10]. For this reason, Sauerbrei and Schumacher [14] proposed the use of bootstrapping methods in combination with automatic backward selection. They ranked the chosen indicators on basis of their selection in the models. Except for the method where only imputation was used, we applied bootstrapping in combination with automatic backward selection in all other methods as proposed by Sauerbrei and Schumacher [14]. However, we extended their method in two ways. First, we included an imputation step for dealing with the missing data. Second, we augmented their method to include estimates of a shrinkage factor.

In our study we found bootstrap corrected *c*-indexes around 0.71 for discrimination in the models developed by the combined methods. Austin and Tu [13] found a *c*-index of 0.82 for a model developed and validated by the use of only the bootstrap method containing variables which were chosen at level of 60%. However, unclear is if they presented the bootstrap corrected *c*-index. Other studies that used the bootstrap had *c*-indexes between 0.70 and 0.80 [10,39], and reported slope values within the range of 0.80 and 0.90, similar to our results. For our models that combined MI and the bootstrap, we found a large decrease in the slope at the threshold of 60% compared to 70%. At 70%, the slope values were 0.79 and 0.80, which decreased to 0.67 and 0.64 at the threshold of 60%. Simultaneously, the number of indicators in these models changed from 11 at the 70% level to 27 and 26 at the 60% level. Steyerberg (2001) [10] demonstrated that it is better to use a more complete model to derive a shrinkage factor to improve the generalization of results to future patients. On basis of this recommendation the *c*-index and the slope among the models in our study with the 70% threshold provides a reasonable trade-off. When a parsimonious model is more important a model that is chosen at a higher inclusion threshold, e.g. 90%, is a good alternative.

In our procedure, identification of strong predictors precede in two steps: first a selection on basis of the p-value, then a selection based on the inclusion frequency. These steps are communicating channels. One strategy is to be fairly flexible in the first step using a p-value of 0.5 and apply a strict variable inclusion frequency level of 90% in step 2. Another strategy is to be strict in step 1 (e.g. take a p-value of 0.157 or 0.05) and take a more lenient value at step 2 (e.g. 70%). Preferably, both routes would produce the same final model, and if this is the case, this will lend credence to the model.

We assumed that the data were missing at random (MAR). It is, by definition, not possible to test the MAR assumption. The prognostic variables that we have included in our study are fairly comprehensive with respect to their importance in low back pain studies. Using all these data in the imputation model makes the MAR assumption plausible, even if the data are not missing at random [6]. It is therefore reasonable to assume that although some variables might be not MAR, this is ignored by the inclusion of other variables in the imputation model when MI is applied [8]. Furthermore, if there are deviations from the MAR assumption in the data set the question is to what extent this affects the final results. Collins et al. [40] showed in a simulation study that an incorrect MAR assumption only had a minor effect on estimates and standard errors in combination with MI. Van Buuren et al. [41] reported in several strongly MAR incompatible models that the negative effects on estimates after MI were only minimal. On basis of these study results we are fairly confident that we have generated valid imputations and that we were able to make reliable inferences from our data. It has been shown in the literature that imputation of outcome variables using the predictors under study minimizes bias in the relationship between predictor and outcome [42,43]. In our data set also some values with respect to the outcome variable were missing. We therefore choose to impute these missing values within the MI algorithm.

Specification of the full imputation model is preferable, but led to computational problems. We followed the guidelines as described by van Buuren et al. [31] and Clark and Altman [9]. These guidelines consist of a number of steps for predictor selection in the context of imputation. Note that such procedures are essentially ad-hoc, and thus open to further research.

A common rule of the thumb states that sample size should be at least 10 times the number of events. In our case, the events per variable (EPV) ratio was 4.4, which according to some would be too low for reliable modeling [44]. Observe however that our methodology takes sample size fully into account and corrects for dangers of overfitting that may result from small samples. Overfitting diagnostics in Table 3 present the effect of sample size on the final model, and may be used to correct the model. Our methodology thus appears to have advantages over other methods if the sample size falls below the EPV > 10 rule.

We did not study the effect of non-linearity or interaction terms under automatic selection techniques. Studying the effects of non-linearity would make the presentation somewhat more complicated. Non-linear effects can be present in all our methods and there is no inherent limitation to main effects models only. Royston and Sauerbrei have shown that it is straightforward to control for non-linear effects by using fractional polynomials within a bootstrapping context [45]. Our method can thus be adjusted to include relevant nonlinearities in the prognostic model. Allowing for interaction makes things more complicated, but there is nothing in our methodology that prevents the use of interactions. When desired, interaction terms can be included by starting from the final main effect of the multivariable model. In principle, the imputation model should also contain the relevant interactions, but the specification of the imputation will become more cumbersome. Not much is known about the strength of the influence of omitting or including interactions on the final inference.

As far as we know, this is the first study that addresses both multiple imputation and sampling variation on the inclusion frequency of prognostic variables. The bootstrap method for investigating the model building is not new, but is still somewhat experimental. Chen and George (1985) [46] described this procedure more than 2 decades ago for the Cox model. Sauerbrei and Schumacher [14], and Augustin [47] extended this method by using the bootstrap to account for uncertainty in model selection as proposed by Buckland [48]. In our study we accounted for uncertainty in selecting a model by means of sampling variation. Sauerbrei and Schumacher [14], and Augustin et al. [47] tested their methods in data sets containing missing values using complete case analyses. Our study provides a more principled alternative.

## Conclusion

Missing data frequently occurs in prognostic studies. Multiple imputation, to handle missing data, and bootstrapping, to select prognostic models, are increasingly applied in prognostic modeling. Both are promising techniques, but both may also complicate the model building process. We showed that it is possible to combine multiple imputation and bootstrapping, thereby accounting for uncertainty in imputations and uncertainty in selecting of models.

## Competing interests

The author(s) declare that they have no competing interests.

## Authors' contributions

MWH participated in the planning and design of the study, the statistical analysis and drafted the manuscript. SVB participated in the planning and design of the study and the statistical analyses. DLK participated in the planning and design of the study. WVM participated in the planning of the study. HCWDV participated in the planning, design and supervision of the study. All authors read and approved the final manuscript.

## Pre-publication history

The pre-publication history for this paper can be accessed here:


